# Effects of blood flow restriction training on cardiometabolic health and body composition in adults with overweight and obesity: a meta-analysis

**DOI:** 10.3389/fphys.2024.1521995

**Published:** 2025-01-17

**Authors:** Hao Kong, Yilin Zhang, Mingyue Yin, Kai Xu, QingGuo Sun, Yun Xie, Olivier Girard

**Affiliations:** ^1^ Athletic Training Institute, Tianjin University of Sport, Tianjin, China; ^2^ School of Athletic Performance, Shanghai University of Sport, Shanghai, China; ^3^ School of Human Sciences (Exercise and Sport Science), The University of Western Australia, Perth, Australia

**Keywords:** vascular occlusion, kaatsu, body fat percentage, systolic blood pressure, obesity

## Abstract

**Objective:**

This meta-analysis aims to evaluate the effects of blood flow restriction (BFR) training on cardiometabolic health and body composition in adults with overweight and obesity.

**Method:**

Following PRISMA guidelines, a systematic search of PubMed (MEDLINE), EMBASE, Web of Science, Cochrane, and Scopus databases was conducted on 15 March 2024. Pooled effects for each outcome were summarized using Hedge’s *g* (*g*) through meta-analysis-based random effects models, and subgroup analyses were used to explore moderators.

**Results:**

A total of 11 studies with 242 participants (Age:32.6 ± 3.6, BMI:27.2 ± 3.5) were included. Regarding cardiometabolic health, BFR training significantly reduced systolic blood pressure (*g* = *0.62* [0.08, 1.16], *p* = 0.02), while no significant differences were observed in maximal oxygen uptake (*g = 0.48* [-0.21, 1.17], *p* = 0.17) or diastolic blood pressure (*g = 0.31* [-0.22, 0.84], *p* = 0.25). Regarding body composition, BFR training significantly reduced body fat percentage (*g* = *0.30* [0.01, 0.58]; *p* = 0.04), while no significant differences (*p* > 0.05) were observed in body weight (*g = 0.14* [-0.14, 0.42]), body mass index (*g = 0.08* [-0.21, 0.38]), waist circumference (*g = 0.13* [-0.28, 0.53]), or waist-to-hip ratio (*g = 0.48* [-0.19, 1.15]). Subgroup analysis revealed no significant difference in improving systolic blood pressure (*g = 0.57* [-0.10, 1.24] vs. *g* = *0.70* [-0.18, 1.59]) and body fat percentage (*g = 0.20* [-0.20, 0.61] vs. *g = 0.45* [-0.05, 0.95]) between BFR resistance training and BFR aerobic training. In all selected studies, the overall risk of bias was categorized as “some concern”. The certainty of evidence for the BFR outcomes was low.

**Conclusion:**

BFR training shows promise in improving cardiometabolic health and body composition, indicating that it may serve as a beneficial, individualized exercise prescription for improving cardiovascular disease risk and fat loss in adults with excess body weight and obesity.

**Systematic Review Registration:**

https://archive.org/details/osf-registrations-uv6jx-v1.

## Introduction

Global obesity rates have surged in recent years, doubling in over 70 countries since 1980, and are projected to reach 57.8% by 2030 (GBD, 2015 Obesity Collaborators et al., 2017). Overweight and obesity are defined by an increased body mass index (BMI), calculated as weight (kg) divided by height (m)^^ 2^ (Nuttall, 2015; Keys et al., 2014). High BMI is strongly associated with various chronic diseases, including cardiovascular disease, diabetes, chronic kidney disease, and musculoskeletal disorders (GBD, 2015 Obesity Collaborators et al., 2017). This contributes to a decreased quality of life and elevated health risks, posing a major global public health challenge that demands urgent attention (Haslam and James, 2005). According to a recently published expert consensus on global fitness trends for 2025, exercise-induced weight loss remains a top priority, ranking as the fourth largest trend. While regular physical activity provides health benefits regardless of weight status, it is essential to incorporate exercise into a long-term, multifaceted approach for effective weight management and maintenance (Newsome et al., 2024).

Numerous studies have shown that exercise can significantly reduce BMI and lower cardiovascular disease risk in adults with overweight and obesity (Wu et al., 2009; Johns et al., 2014; Oppert et al., 2021). For example, high-intensity interval training is a time-efficient strategy that can induce physiological and psychological adaptations related to improved cardiometabolic health in adults with overweight and obesity (Batrakoulis et al., 2021; Poon et al., 2024; Yin et al., 2024a; Yin et al., 2023). Combining resistance training with aerobic or endurance training not only significantly improves cardiometabolic health in this population but also enhances fat loss (Garber et al., 2011; Oppert et al., 2023). Among them, combined aerobic exercise and diet play a key role in various aspects of cardiometabolic health, including blood pressure, lipid homeostasis, glucose homeostasis, adipose tissue dysfunction, and chronic inflammation in adults with obesity (Al-Mhanna et al., 2023). In addition, aerobic training and high-intensity interval training can reduce body weight (BW), body fat percentage (BF%), and abdominal visceral fat, while resistance training helps preserve fat-free mass (FFM) (Alberga et al., 2011). The latest meta-analysis shows that combined training is the most effective approach for improving cardiometabolic health outcomes in these populations (Batrakoulis et al., 2022).

From a practical perspective, adults with overweight and obesity may be at increased risk of sports injuries due to prolonged physical inactivity, participation in intense activities (e.g., high-intensity interval training), or unfamiliar forms of exercise (e.g., resistance training) (Kim and Yoon, 2021). Therefore, this population must adopt appropriate and progressive training methods to ensure both safety and effectiveness. Blood flow restriction (BFR) training, which involves applying external pressure to the proximal part of the upper or lower limb using a cuff or inflatable device during exercise (Lorenz et al., 2021), offers a potential solution. The term BFR training used in the following text refers to blood flow restriction combined with both aerobic and resistance training. Research suggests that BFR combined with low-intensity resistance or aerobic exercise can mimic the effects of high-intensity exercise, providing comparable benefits in cardiometabolic health and body composition (Bradley et al., 2023; Patterson et al., 2019). For example, BFR combined with resistance training or aerobic training can significantly improve muscle mass and maximal oxygen uptake (V̇O_2_max) (Kim et al., 2021; Yin et al., 2025). In addition, BFR combined with low-load resistance training is more effective at increasing muscle mass than low-load resistance training alone (Hughes et al., 2017).

While BFR combined with low-intensity resistance or aerobic exercise has proven benefits for improving cardiometabolic health and body composition in rehabilitation and healthy populations, its effects on adults with overweight and obesity remain less clear. A meta-analysis showed that BFR training effectively reduced BMI (mean difference [MD] = -0.77, *p* = 0.02) in these individuals, without distinguishing between exercise modes (Sun, 2022). But no significant changes were observed in body weight (MD = −1.32, *p* = 0.15), waist circumference (MD = −0.73, *p* = 0.19), and body fat percentage (MD = −0.66, *p* = 0.10), as these results did not reach statistical significance. However, with only four studies included, the meta-analysis may have lacked sufficient statistical power, potentially affecting the accuracy of the findings. In addition, the analysis was influenced by a few heavily weighted studies (e.g., body fat percentage at 84.9%, BMI at 62.4%), which may have introduced bias into the pooled results.

Currently, no systematic review or meta-analysis has specifically examined the effects of BFR training on cardiometabolic health in adults with overweight and obesity. Furthermore, studies comparing the differential effect of resistance or aerobic exercise with BFR on cardiometabolic health remain lacking. This research gap limits a comprehensive understanding of the health-promoting effects of BFR training in these populations. Evidence shows that, compared to aerobic exercise without BFR, aerobic exercise with BFR significantly increases V̇O_2_max by 12.6% (Adalberto, 2019) and reduces systolic blood pressure (SBP) by 2.8% (Ferreira Junior et al., 2019). In contrast, while resistance exercise with BFR demonstrates an effect in lowering SBP (Su et al., 2024), some studies report no significant improvement in cardiometabolic health (Bradley et al., 2023; Libardi et al., 2015a). These findings suggest that aerobic exercise with BFR may provide greater benefits for cardiometabolic health than resistance exercise with BFR. However, due to the limited number of studies and inconsistent results, a meta-analysis is urgently needed to systematically review and synthesize the current evidence, clarifying the specific effects and relative advantages of resistance exercise with BFR and aerobic exercise with BFR on cardiometabolic health in adults with overweight and obesity.

Therefore, the present meta-analysis focuses on the effects of BFR training on cardiometabolic health and body composition in adults with overweight and obesity, and examines how training protocol parameters may influence these outcomes.

## Methods

This review follows the preferred reporting items for systematic reviews and meta-analyses (PRISMA) guidelines (Page et al., 2021), and is registered in the Open Science Framework (https://doi.org/10.17605/OSF.IO/UV6JX).

### Literature search

Literature searches were performed in PubMed, Scopus, Embase, Web of Science and Cochrane. Keywords were drawn from controlled vocabularies (e.g., Medical Subject heading: MeSH). Search terms included: (“Blood flow restriction” or “Blood flow occlusion” or “Kaatsu” or “Vascular occlusion” or “Restricted blood flow” or “Occlusion training” or “Occluded training”)and (“BMI” or “waist circumference” or “hip circumference or” waist to “hip ratio” or “resting heart rate” or “%body fat” or “lean body mass” or “fat mass” or “Muscle mass” or “body weight” or “free fat mass”) and (“blood pressure” or “V̇O_2_max” or “fitness” or “cardiorespiratory fitness” or “V̇O_2_peak”) and (“Human” (MeSH)). Publications were searched from the inception of each database until 15 March 2024. A reference list of articles that met the inclusion and exclusion criteria is also provided. Two authors (KH and ZYL) conducted the initial search to remove duplicates and screened the papers according to the inclusion criteria. Any disagreements were resolved through discussion with a third author (SQG).

### Eligibility criteria

The PICOS (Participants, Interventions, Comparators, Outcomes, and Study Design) framework was applied to assess the eligibility of the studies. Peer-reviewed articles were excluded if they involved non-human experiments or recruited unhealthy participants. The specific inclusion and exclusion criteria are listed below:

#### Population

The study recruited healthy adults with overweight (BMI: 25.0–29.9) or obesity (BMI: ≥30), people aged 18 or older, regardless of sex or country. Athletes or trained adults were excluded.

#### Intervention

BFR is a technique that restricts blood flow to the muscles, typically by applying light pressure using specialized bands or cuffs on the upper or lower limbs. We included both resistance training and aerobic exercise because our primary focus is on the broad application of blood flow restriction training for cardiometabolic health in adults with overweight and obesity. Therefore, we did not limit ourselves to a specific type of exercise but aimed to explore the broader effects of blood flow restriction. We will address this potential confusion through subgroup analyses. Resistance training is defined as exercises involving external resistance (e.g., squats (Kim et al., 2021; Su et al., 2024), elbow flexion (Su et al., 2024; Libardi et al., 2015b; Farzaneh Hesari et al., 2018; da Silva et al., 2020), knee extension (Kim et al., 2021; Farzaneh Hesari et al., 2018; da Silva et al., 2020), bench press (Kim et al., 2021),deadlift (Bradley et al., 2023)). Aerobic exercise is defined as low-intensity, sustained activities (e.g., walking (Adalberto, 2019), cycling (Chen et al., 2022), rowing (Bradley et al., 2023)). During aerobic or resistance training, participants used commercially available BFR devices. Studies needed to provide sufficient data to calculate effect sizes and assess the chronic effects.

#### Comparison

The intervention group added BFR to aerobic training, resistance training, or a combination of both, while the control group did not. The primary difference between the intervention and control groups throughout the study was the use of BFR. Baseline demographic characteristics between the groups were not different to ensure an accurate assessment of the intervention’s effects.

#### Outcomes

The study evaluated the effect of BFR training on at least one of the following outcome measures, comparing post-intervention results to baseline/pre-training values: 1) V̇O_2_max: assessed using gas-exchange data and heart rate during progressive exercise tests including treadmill (Adalberto, 2019; Libardi et al., 2015b), stationary rowing machine (Bradley et al., 2023), or gradient cycling (Li et al., 2022). 2) Heart rate: measured using a Polar chest strap. 3) Blood pressure: SBP and Diastolic Blood Pressure (DBP) were measured at rest and before and after exercise with automated electronic sphygmomanometer or mercury sphygmomanometers. 4) Body composition: BW, BMI, FFM, Fat Mass (FM), BF%, and MM were measured using body composition analyzers (DX-200, Inbody 720, and Maltron BF-906), dual-energy X-ray absorptiometry (DEXA), and Waist Circumference (WC) was measured with inelastic measuring tape for Waist-to-Hip Ratio (WHR) calculation.

#### Study design

The analysis included randomized controlled trials with clear descriptions of the intervention and outcome measures.

### Risk of bias assessment

The Cochrane Risk of Bias 2 (RoB) tool was used to assess studies that met the inclusion criteria. It assesses five potential sources of bias: 1. randomization process; 2. deviations from the intended intervention (including distributional effects and adherence); 3. missing outcome indicators; 4. outcome measures; and 5. selection of reported outcomes. Each domain was rated as “yes”, “probably yes”, “probably no”, or “no”. According to the Cochrane guidelines (the “Cochrane Handbook for Systematic Reviews of Interventions”), the overall risk of bias is categorized as “high” (if at least one domain indicates high risk), “some concern” (if at least one domain indicates concern but no high risk), and “low” (if all domains show low risk).

### Data extraction and conversion

We extracted mean, standard deviation, and sample size data for each group before and after the intervention. The effects were summarized as differences between pre- and post-intervention outcomes. Mean differences were calculated by subtracting pre-intervention means from post-intervention means for each group. For studies reporting standard errors (SEM), these were converted to standard deviation (SD) using the formula SD = SEM $\sqrt{N}$ , where SD is the standard deviation, SEM is the standard error of the mean, and N is the sample size.

The SD of the difference in means was then calculated as follows:

$${SD}_{diff}=\sqrt{{SD}_{pre}^{\;2}+{SD}_{post}^{\;2}-2r\times {SD}_{pre}\times {SD}_{post}}$$

where SD_diff_ is the standard deviation of the pre- and post-intervention differences, SD_pre_ and SD_post_ are the standard deviations for pre- and post-intervention measures, respectively, and r is the correlation coefficient between pre- and post-intervention measurements.

As the original studies did not report Pearson’s correlation coefficients (r) for pre- and post-intervention outcomes, we referred to meta-analyses with similar results and chose r = 0.5 for body composition indicators and r = 0.6 for cardiometabolic indicators for sensitivity analyses (Table 1).

**TABLE 1:** Sensitivity analysis.

	Correlation coefficient	Random effects model		z	p-value	Tau^2^	Quantifying heterogeneity		95%-CI	Test of heterogeneity		
		Hedge’g	95%-CI				95%-CI	I^2^(%)		Q	d.f	p-value
Cardiometabolic health	r-0.50	0.2738	[0.0173; 0.5303]	2.09	0.0364	0	[0.0000; 0.0126]	0.00%	[0.0%; 60.20%]	3.46	10	0.9683
	r-0.55	0.2875	[0.0307; 0.5443]	2.19	0.0282	0	[0.0000; 0.0336]	0.00%	[0.0%; 60.20%]	3.82	10	0.955
	r-0.60	0.3035	[0.0464; 0.5060]	2.31	0.0207	0	[0.0000; 0.0598]	0.00%	[0.0%; 60.2%]	4.27	10	0.9346
	r-0.65	0.3224	[0.0649; 0.5799]	2.45	0.0141	0	[0.0000; 0.0934]	0.00%	[0.0%; 60.2%]	4.82	10	0.9026
	r-0.70	0.3455	[0.0874; 0.6035]	2.62	0.0087	0	[0.0000; 0.1379]	0.00%	[0.0%; 60.2%]	5.55	10	0.8515
	r-0.75	0.3743	[0.1156; 0.6331]	2.84	0.0046	0	[0.0000; 0.1998]	0.00%	[0.0%; 60.2%]	6.54	10	0.7685
	r-0.80	0.4118	[0.1521; 0.6716]	3.11	0.0019	0	[0.0000; 0.2915]	0.00%	[0.0%; 60.2%]	7.95	10	0.6341
	**r-0.85**	**0.4636**	**[0.2023; 0.7250]**	**3.48**	**0.0005**	**< 0.0001**	**[0.0000; 0.4413]**	**0.00%**	**[0.0%; 60.8%]**	**10.14**	**10**	**0.4282**
	r-0.90	0.5425	[0.2335; 0.8516]	3.44	0.0006	0.0711	[0.0000; 0.7302]	28.70%	[0.0%; 64.9%]	14.03	10	0.1715
Body composition	**r-0.50**	**0.1643**	**[0.0321; 0.2965]**	**2.44**	**0.0149**	**0**	**[0.0000; 0.0199]**	**0.00%**	**[0.0%; 38.3%]**	**22.23**	**34**	**0.9398**
	r-0.55	0.1721	[0.0397; 0.3.44]	2.55	0.0108	0	[0.0000; 0.0380]	0.00%	[0.0%; 38.3%]	24.38	34	0.8879
	r-0.60	0.1811	[0.0486; 0.3136]	2.68	0.0074	0	[0.0000; 0.0605]	0.00%	[0.0%; 38.3%]	27.01	34	0.797
	r-0.65	0.1917	[0.0590; 0.3244]	2.83	0.0046	0	[0.0000; 0.0891]	0.00%	[0.0%; 38.3%]	30.29	34	0.6499
	r-0.70	0.2045	[0.0714; 0.3375]	3.01	0.0026	0.0003	[0.0000; 0.1267]	1.50%	[0.0%; 39.2%]	34.51	34	0.4434
	r-0.75	0.2197	[0.0758; 0.3635]	2.99	0.0028	0.025	[0.0000; 0.1783]	15.30%	[0.0%; 44.4%]	40.14	34	0.2166
	r-0.80	0.2404	[0.0821; 0.3987]	2.98	0.0029	0.0616	[0.0000; 0.2532]	29.30%	[0.0%; 53.5%]	48.07	34	0.0555
	r-0.85	0.2706	[ 0.0919; 0.4494]	2.97	0.003	0.1206	[0.0268; 0.3725]	43.50%	[15.5%; 62.2%]	60.13	34	0.0038
	r-0.90	0.3197	[ 0.1082; 0.5312]	2.96	0.0031	0.231	[0.1009; 0.5944]	58.00%	[38.9%; 71.1%]	80.98	34	< 0.0001

Number of studies combined: k_Cardiometabolic health_ = 7, k_Body composition_ = 10, Inverse variance method, Restricted maximum-likelihood estimator for tau², Q-profile method for confidence interval of tau² and tau; Hedge’s, the effect size indicators used in the pooled; 95%CI, 95% confidence interval; P-value, statistically significant P values for pooled results; I^2^, quantitative indicators of heterogeneity; Q, Heterogeneity statistic; d.f, Degrees of Freedom.

### Statistical analysis

Statistical analyses were performed using the ‘meta’ and ‘metafor’ packages of the R statistical software (Version.4.3.3). Meta-analyses utilized the general inverse variance merging method, with effect sizes combined using the random effects model of the DerSimonia-Laird method to summarise the effects of BFR combined with resistance or aerobic training on body composition and cardiometabolic fitness compared to controls (DerSimonian and Laird, 1986). Effects were expressed as Standardized Mean Difference (SMD) and estimated Hedge’g, categorised as *negligible* (0.2), *small* (0.2–0.5), *medium* (0.5–0.8), and *large* (>0.8). Statistical significance was set at *p* < 0.05. Heterogeneity was assessed using I^2^, with thresholds of 25%, 50%, 75%, and >75% indicating *low*, *medium*, *high* and *very high* thresholds, respectively. We calculated 95% confidence intervals (CI) and determined prediction intervals (PIs) using t-distribution to account for heterogeneity (Nagashima et al., 2018). Studies with CI that did not overlap with the combined effect CI were considered statistical outliers. The impact of individual studies was assessed through impact analyses using the leave-one-out method.

We conducted a separate meta-analysis to evaluate the effects of BFR training on cardiometabolic health and body composition in adults with overweight and obesity. Due to the limited number of studies, we subsequently performed subgroup analyses based on exercise modality to compare the effects of aerobic or resistance exercise combined with BFR on cardiometabolic health and body composition. Additionally, for body composition, considering that only one study involved sprint training, the subgroup analysis was restricted to aerobic exercise with BFR and resistance exercise with BFR.

### Quality of evidence

The quality of scientific evidence was assessed using the GRADE manual recommendations (Schünemann et al., 2019). The GRADEpro GDT software was employed to rate the quality of evidence across four domains: high, moderate, low, and very low. The evaluation of BFR training interventions for improving body composition and cardiometabolic health considered the risk of bias, inconsistency of results, indirectness of evidence, imprecision of results, and publication bias. Initially, evidence was rated as having a high degree of certainty but was downgraded according to the following criteria: (GBD, 2015 Obesity Collaborators et al., 2017): Risk of bias: downgraded one level for some concerns and two levels for high risk; (Nuttall, 2015); Indirectness: downgraded one level for indirectness (e.g., inconsistent populations, interventions, comparators, and outcomes) and two levels if the indirectness was widespread; (Keys et al., 2014); Publication bias: downgraded one level if publication bias was suspected, as indicated by Egger’s test; (Haslam and James, 2005); Inconsistency: downgraded one level for high inter-study heterogeneity (I^2^ > 50%) or poor overlap of confidence intervals; (Newsome et al., 2024); Imprecision: downgraded one level for sample sizes of <800 or non-significant results, and two levels if both criteria for imprecision applied.

## Results

### Study selection

**FIGURE 1:**
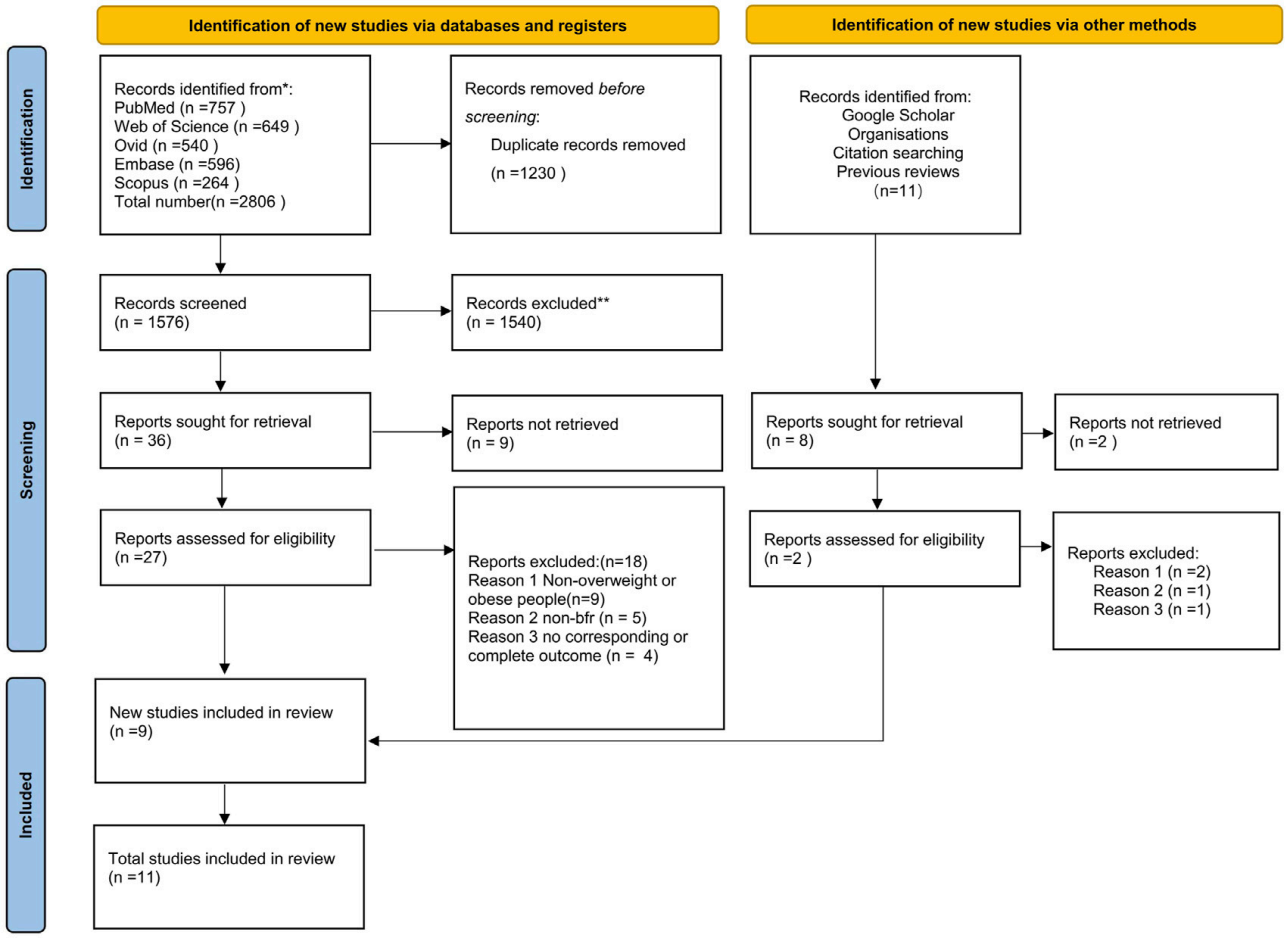
PRISMA flow diagram for included and excluded study.

An initial database search identified 2806 publications. After screening, 11 papers were deemed eligible for inclusion in the meta-analysis (Figure 1).

### Study characteristics

The studies included in this systematic review spanned the period 2015–2024. A total of 242 adults with overweight and obesity were assessed, including 97 males and 74 females, with 71 subjects having unspecified gender (Age: 32.6 ± 3.6; BMI: 27.2 ± 3.5). Detailed descriptions of participant characteristics, BFR cuff position and pressure, and interventions are provided in Tables 2, 3.

**TABLE 2:** Characteristics of studies on blood flow restriction training for cardiometabolic health in adults with overweight and obesity.

Study	Participant Characteristics				BFR Details		Protocols						
	Age	BMI	M/F	N	Position	Pressure	Intervention	Intensity	Sets × Reps or Span	Interval	Frequency	Duration(weeks)	Results
Libardi et al. (2015a)	64.7 ± 4.1	26.6 ± 3.0	n/a	10	PT	50%AOP; 67±8.0mmHg	LI-RT	20-30%1-RM	4 × 15-30	1 min	4 t/wk	12	V̇O_2_max ↔
Ferreira Junior et al. (2019)	51.9 ± 3.7	28.7 ± 3.0	11/0	11	PT	50-100mmHg	LI-AT	6 km/h	5 × 3 min	1 min	3 t/wk	6	SBP ↓; DBP ↔
Su et al. (2024)	20-24	25.4 ± 3.8	9/0	9	PT	140-200mmHg	LI-RT	30%1-RM	4 × (15-30)	30-60 s	5 t/wk	12	SBP↓; DBP ↔
Bradley et al. (2023)	30.2 ± 7.7	27.08 ± 7.5	6/3	9	PT	80%AOP	LI-AT+LI-RT	40% maximal power output; 30% 1-RM	5 × (1-2min);4 × (10-20)	30 s	2 t/wk	4	V̇O_2_max ↔
Adalberto (2019)	51.9 ± 3.5	28.6 ± 2.8	14/0	14	PT	80-100mmHg	LI-AT	6 km/h	5 × 3 min	1 min	3 t/wk	6	V̇O_2_max↑; HR ↓
Li et al. (2022)	22.1 ± 2.0	26.5 ± 1.4	n/a	17	PT	40%AOP	HIIT	85% V̇O_2_max	3 × 3min	3 min	2 t/wk	12	V̇O_2_max ↔
Farzaneh Hesari et al. (2018)	18-24	27.8 ± 3.0	0/9	9	PT,PUL	<70%AOP	LI-RT	30%1RM	4 × (15-30)	1-4min	3 t/wk	8	SBP ↔; DBP ↔

n: sample size of the BFR, PT: proximal thigh, PUL: proximal upper limb, AOP: arterial occlusion pressure, Reps: repetitions, t: times, wk: week, h:hours, ±:mean and SD.

**TABLE 3:** Characteristics of studied on blood flow restriction training for body composition in adults with overweight and obesity.

Study	Participant Characteristics				BFR Details		Protocols						
	Age	BMI	M/F	N	Position	Pressure	Intervention	Intensity	Sets × Reps or Span	Interval	Frequency	Duration(weeks)	Results
Ferreira Junior et al. (2019)	51.9 ± 3.7	28.7 ± 3.0	11/0	11	PT	50-100mmHg	LI-AT	6 km/h	5 × 3 min	1 min	3 t/wk	6	BW ↔
Chen et al. (2022)	20.3 ± 1.1	30.1 ± 0.9	18/0	18	PT	160–200mmHg	LI-AT	40% V̇O_2_max	3 × 15 min	n/a	2 t/wk	12	BMI ↓; WC ↓;BW ↓;FFM(kg) ↔;FM ↓;BF ↓
Su et al. (2024)	20-24	25.4 ± 3.8	n/a	9	PT	140-200mmHg	LI-RT	30%1-RM	4 × (15-30)	30-60 s	5 t/wk	12	Lean Mass ↑; BF ↓; WHR ↓;BW ↔;FM ↔;MM ↑
Adalberto et al. (2019)	51.9 ± 3.5	28.6 ± 2.8	14/0	14	PT	80-100mmHg	LI-AT	6 km/h	5 × 3 min	1 min	3 t/wk	6	BW ↔;BMI ↔;BF ↔;FM ↔;FFM(kg) ↔;FFM(%) ↔
da Silva et al. (2020)	25.5 ± 2.2	28.5 ± 2.4	15/0	15	PT,PUL	20-40mmHg	LI-RT	30%1-RM	4 × 23	2 min	3 t/wk	8	BMI ↔; WC ↔; BF ↔;BW ↔;FFM(%) ↔
Li et al. (2022)	22.1 ± 2.0	26.5 ± 1.4	n/a	17	PT	40%AOP	HI-S	85% V̇O_2_max	3 × 3 min	3 min	2 t/wk	12	BF ↓;FM ↔
Farzaneh Hesari et al. (2018)	18-24	27.8 ± 3.0	0/9	9	PT,PUL	<70%AOP	LI-RT	30%1-RM	4 × (15-30)	1-4 min	3 t/wk	8	WHR ↔;BMI ↔;BW ↔
Kim et al. (2021)	22.3 ± 1.0	22.3 ± 3.3	0/9	9	PT,PUL	n/a	LI-RT	40%1-RM	3 × (14-18)	n/a	3 t/wk	5	BW ↔;FM ↔;BMI ↔; BF ↔;MM ↔
Mohammadiyan (2021)	27.4 ± 5.2	27.6 ± 2.7	0/15	15	PUL	n/a	LI-RT	20%-30%1-RM	3 × (20-50)	60-90 s	3 t/wk	6	BW ↓;BMI ↓;BF ↓;WC ↓

n: sample size of the BFR, PT: proximal thigh, PUL: proximal upper limb, AOP: arterial occlusion pressure, Reps: repetitions, t: times, wk: week, h:hours, ±:mean and SD.

### Risk of bias

**FIGURE 2:**
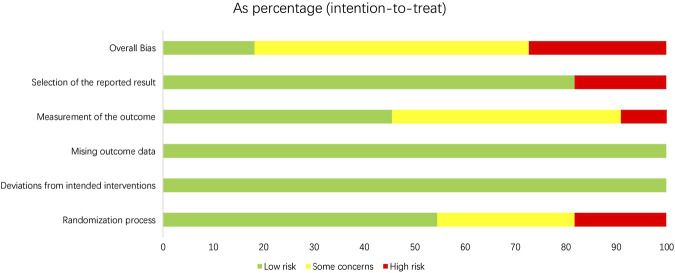
Overall risk of bias.

**FIGURE 3:**
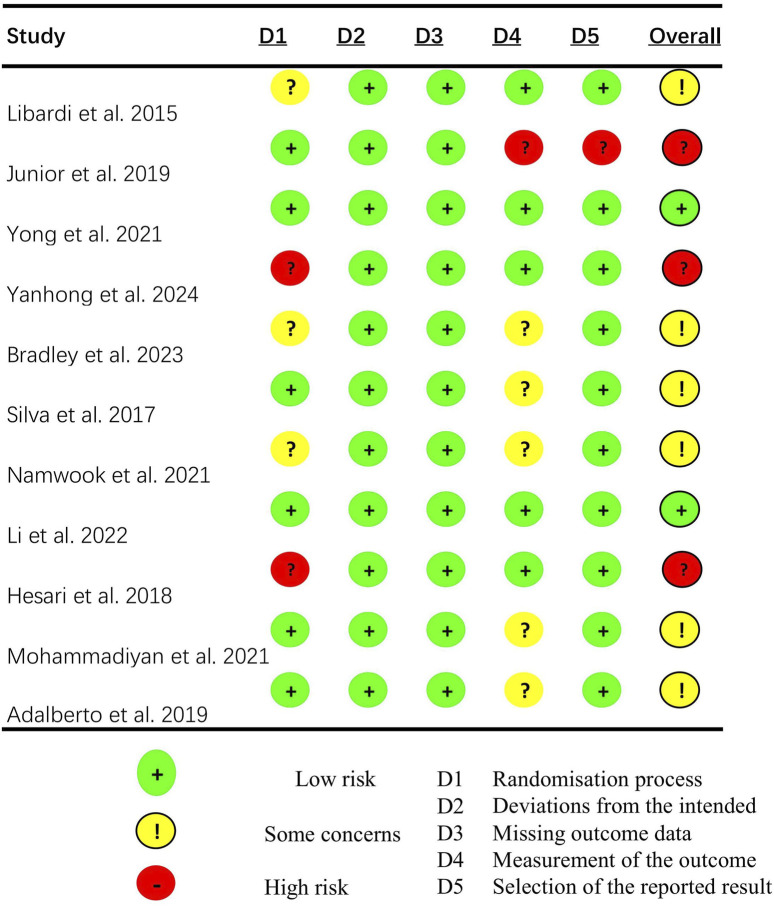
Risk-of-bias assessment.

In all selected studies, the overall risk of bias was categorized as “some concern”. According to the Rob two tool, a reduced risk of bias was noted concerning missing outcome data, while the selection and randomization processes for reporting outcomes were areas of increased risk in most studies (Figures 2, 3).

### Effects of BFR training vs. control

Nine studies assessed the effects of BFR training (Three studies on aerobic exercise, five studies on resistance exercise, and one study on sprinting). on body composition compared to controls, and seven studies assessed its effects on cardiometabolic health (Two studies on aerobic exercise, three studies on resistance exercise, one study on combined aerobic and resistance exercise, and one study on sprinting). All participants were overweight and obesity.

### Cardiometabolic health

**FIGURE 4:**
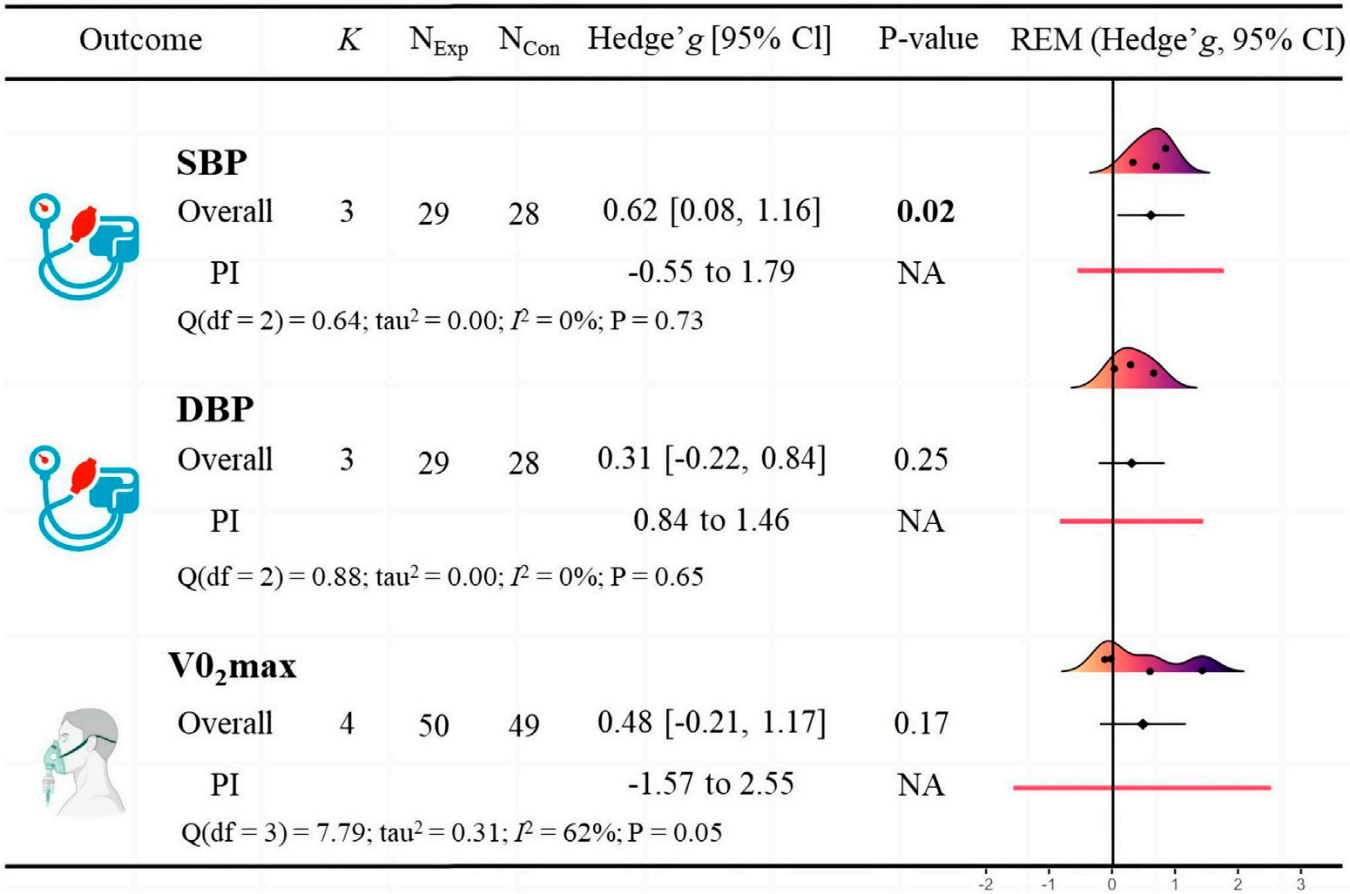
Effects of blood flow restriction training on cardiometabolic health in adults with overweight and obesity.

**FIGURE 5:**
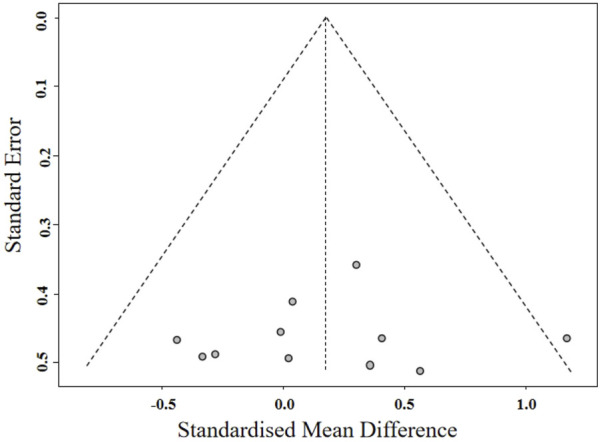
Funnel plot of cardiometabolic health.

A meta-analysis of studies on cardiometabolic health indicators is shown in Figure 4. The results indicated that BFR training significantly improved SBP (*g* = *0.62* [0.08, 1.16], *p* = 0.02). Subgroup analysis revealed no significant difference in improving systolic blood pressure (*g = 0.57* [-0.10, 1.24] vs. *g = 0.70* [-0.18, 1.59]) between BFR resistance training and BFR aerobic training (Table 4). The meta-analysis found no statistically significant effect of BFR training on DBP (*g = 0.31* [-0.22, 0.84], *p* = 0.25), V̇O_2_max (*g = 0.48* [-0.21, 1.17], *p* = 0.17). In addition, Egger’s test showed no significant risk of publication bias (*p* = 0.865; Figure 5).

**TABLE 4:** The subgroup analysis of BFR training on SBP.

		BFRn	CONn	K	ES	95%-CI	Q	df	P_diff_
Exercise Mode	AT	11	10	1	0.7045	[-0.1836; 1.5925]	0.06	1	0.8145
	RT	18	18	2	0.5710	[-0.1023; 1.2444]			

AT: Aerobic Training RT: Resistance Training BFRn: Sample size of BFR group; CONn: Sample size of the Control group; K: Number of studies; ES: Effect Size; 95%-CI: 95% Confidence Interval; Q: Heterogeneity statistic; df: Degrees of Freedom; P_diff_: P-value for difference.

### Body composition

**FIGURE 6:**
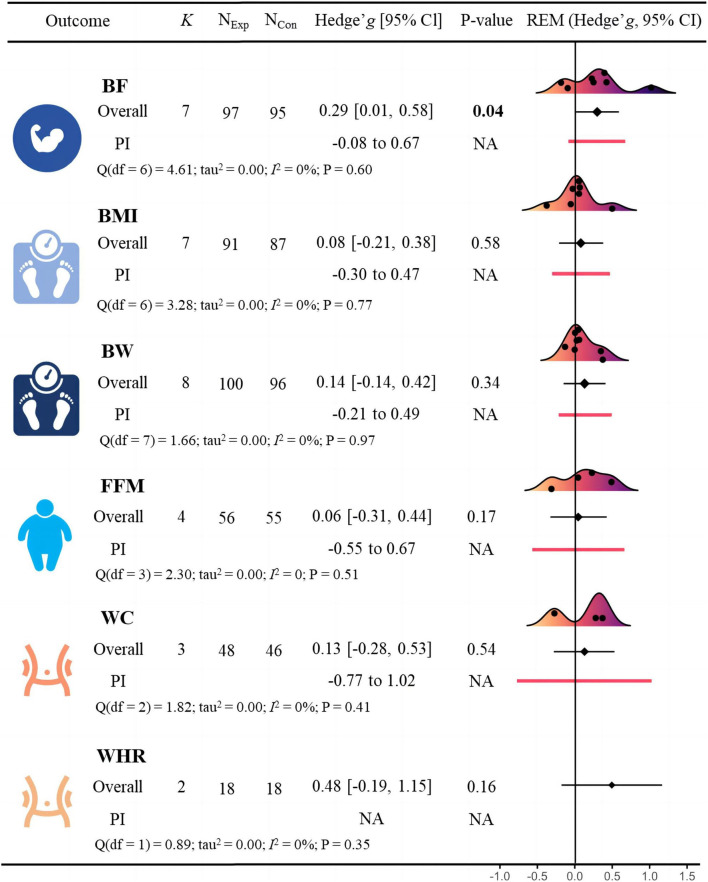
Effects of blood flow restriction training on body composition in adults with overweight and obesity.

**FIGURE 7:**
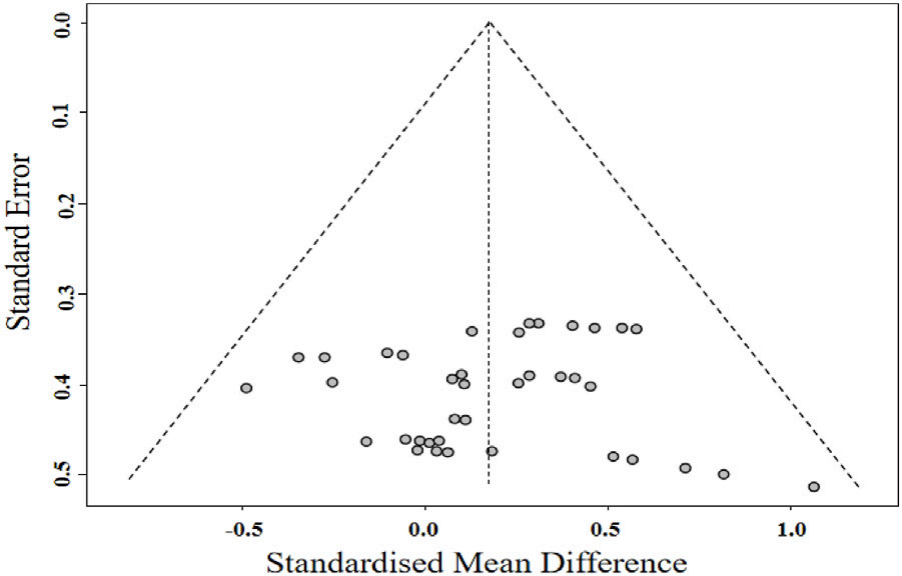
Funnel plot of body composition.

A meta-analysis of studies on body composition indicators is displayed in Figure 6. The meta-analysis revealed that BFR training had a significant positive effect on BF (*g* = *0.30* [0.01, 0.58], *p* = 0.04). Subgroup analysis revealed no significant difference in improving body fat percentage (*g = 0.20* [-0.20, 0.61] vs. *g = 0.45* [-0.05, 0.95]) between BFR resistance training and BFR aerobic training (Table 5). The meta-analysis found no statistically significant effect of BFR training on BMI (*g = 0.08* [-0.21, 0.38], *p* = 0.58), BW (*g = 0.14* [-0.14, 0.42], *p* = 0.34), FFM (*g = -0.26* [-0.79, 0.27]. *p* = 0.17), WC (*g = 0.13* [-0.28, 0.53], *p* = 0.54), and WHR (*g = 0.48* [-0.19, 1.15], *p* = 0.16). Additionally, Egger’s test showed no significant risk of publication bias (*p* = 0.857; Figure 7).

**TABLE 5:** The subgroup analysis of BFR training on BF%.

		BFRn	CONn	K	ES	95%-CI	Q	df	P_diff_
Exercise Mode	AT	32	31	2	0.4507	[-0.0510; 0.9523]	0.49	1	0.4841
	RT	48	46	4	0.2053	[-0.2067; 0.6173]			

AT: Aerobic Training; RT: Resistance Training; BFRn: Sample size of BFR group; CONn: Sample size of the Control group; K: Number of studies; ES: Effect Size; 95%-CI: 95% Confidence Interval; Q: Heterogeneity statistic; df: Degrees of Freedom; P_diff_: P-value for difference.

### Certainty of evidence

The certainty of evidence for the BFR outcomes was low, based primarily on limited sample size for comparison and Risk of study bias is “some concerns”.

## Discussion

To our knowledge, this is the first systematic review and meta-analysis to assess the effects of BFR training on cardiometabolic health and body composition in adults with overweight and obesity. Our main findings indicate BFR training improved SBP and body fat percentage, while showing limited potential on V̇O_2_max, diastolic blood pressure, body weight, BMI, waist circumference, fat-free mass, waist-to-hip ratio, or muscle mass. Additionally, the effects of aerobic exercise with BFR on cardiometabolic health and body composition were not different compared to those with resistance exercises with BFR.

### Effect of BFR training on cardiometabolic health

Our meta-analysis found that low-intensity BFR training significantly reduced SBP in adults with overweight and obesity (*g* = *0.62* [0.08, 1.16]). Further subgroup analysis revealed no significant difference in improving systolic blood pressure (*g = 0.57* [-0.10, 1.24] vs. *g =* 0.70 [-0.18, 1.59]) between BFR resistance training and BFR aerobic training; however, this difference was not statistically significant. Interestingly, recent research has shown that resistance training results in a greater improvement in SBP (10.2%) compared to aerobic training (1.9%) in adults with overweight and obesity (Batrakoulis et al., 2022). Additionally, other studies have found that resistance training significantly improved diastolic blood pressure (SMD = −0.33) in individuals with obesity, but no significant difference was observed in systolic blood pressure (SMD = −0.04) (AL-Mhanna et al., 2024). This difference may reflect the distinct physiological mechanisms underlying the different training modalities. It is also important to note that the effect of BFR combined with aerobic exercise on SBP was evaluated in only one study, which may limit the statistical power and robustness of this finding.

High SBP is a major modifiable risk factor for cardiovascular disease (Lewington and Qizilbash, 2002) and is associated with increased incidence and mortality from cardiovascular conditions (Rutan et al., 1988; Stamler et al., 1993; Sesso et al., 2000). In addition, BMI is nearly linearly related to both SBP and DBP across different populations (Hall, 2003), suggesting a high prevalence of hypertension among adults with overweight and obesity. While not all adults with overweight and obesity suffer from hypertension, weight gain shifts blood pressure distribution to higher levels, increasing the risk of developing hypertension (Hall et al., 2015).

Exercise is known to effectively lowers blood pressure (AL-Mhanna et al., 2024; Arroll and Beaglehole, 1992; Fagard, 1993). Our meta-analysis suggests that BFR combined with low-intensity resistance or aerobic exercise may enhance this antihypertensive effect by providing additional stimuli to the body. Specifically, BFR training increases venous pressure, which can alter venous compliance, vessel wall tone, and endothelial function (Stamler et al., 1993). These changes lead to increased blood flow and shear stress, triggering positive vascular adaptations such as increased levels of vascular endothelial growth factor and endothelial-type nitric oxide synthase, thereby increasing nitric oxide production. The increase in nitric oxide helps dilate blood vessels, thereby lowering blood pressure (Rutan et al., 1988). Although BFR training can cause temporary increases in heart rate and blood pressure during training, long-term BFR training has been shown to more effectively lowers SBP compared to equivalent intensity exercise without external limb compression (Hackney et al., 2012). The significant reduction in SBP observed in our study suggests that BFR training provides an effective, lower-intensity exercise option for adults with overweight and obesity, potentially reducing the exercise burden while improving cardiovascular health. Therefore, BFR training may represent a valuable strategy for preventing hypertension in these populations.

Moreover, existing research has shown that even a modest decrease in systolic blood pressure of 2–3 mmHg is sufficient to significantly reduce the occurrence of cardiovascular events (Elliott, 1991), and a reduction of 4 mmHg or more is expected to lead to a 5%–20% decrease in cardiovascular disease mortality (Adler et al., 2014). Therefore, the findings of our study (a reduction in systolic blood pressure of 3.38–9.28 mmHg) have important clinical significance, demonstrating the potential of BFR training as an effective low-intensity exercise option for adults with overweight and obesity, which not only reduces the exercise burden but also significantly improves cardiovascular health.

However, despite the significant effects of BFR training on blood pressure regulation, its safety remains a topic of debate. A narrative review by Spranger et al. suggests that excessive vascular occlusion induced by the cuff during BFR training may lead to overactivation of muscle reflexes (e.g., activation of type IV and III afferent fibers), which in turn may trigger excessive sympathetic nervous system activity, resulting in increased cardiac function, blood pressure, and vascular resistance (Spranger et al., 2015). Therefore, based on the existing literature, it is recommended that healthcare providers set the restriction pressure during BFR training at 40%–60% of each participant’s arterial occlusion pressure to ensure that no participant experiences unnecessary health risks due to excessively high pressure (Mouser et al., 2017).

The meta-analysis showed no statistically significant effect of BFR training on V̇O_2_max (*g = 0.48* [-0.21, 1.17]). V̇O2max, a skey indicator of cardiorespiratory fitness, typically requires longer periods of moderate-to high-intensity aerobic exercise to show improvement (Swain and Franklin, 2006; Blair et al., 2001; Wenger and Bell, 1986). Engaging in sufficient and regular aerobic or resistance exercise can also reduce the risk of cardiovascular disease and high BMI, as well as improve insulin resistance (Badri Al-mhanna et al., 2024). The low intensity and short duration of BFR training may be insufficient to induce significant cardiorespiratory adaptations. However, the lack of significant effect on V̇O_2_max does not diminish the clinical value of BFR training. It remains a safe and potentially beneficial option for adults with overweight and obesity who may not be suited for high-intensity training.

While BFR training significantly improved SBP, its effect on V̇O_2_max remains unclear. Future research should focus on optimizing Aerobic or resistance exercises with BFR protocols and explore the potential of combining different exercise modalities with blood flow restriction for improving cardiometabolic health and body composition in adults with overweight and obesity. Increasing the intensity and duration of BFR training interventions may help determine their impact on V̇O_2_max and further enhance the application of BFR training for adults with overweight and obesity.

### Effects of BFR training on body composition

**FIGURE 8:**
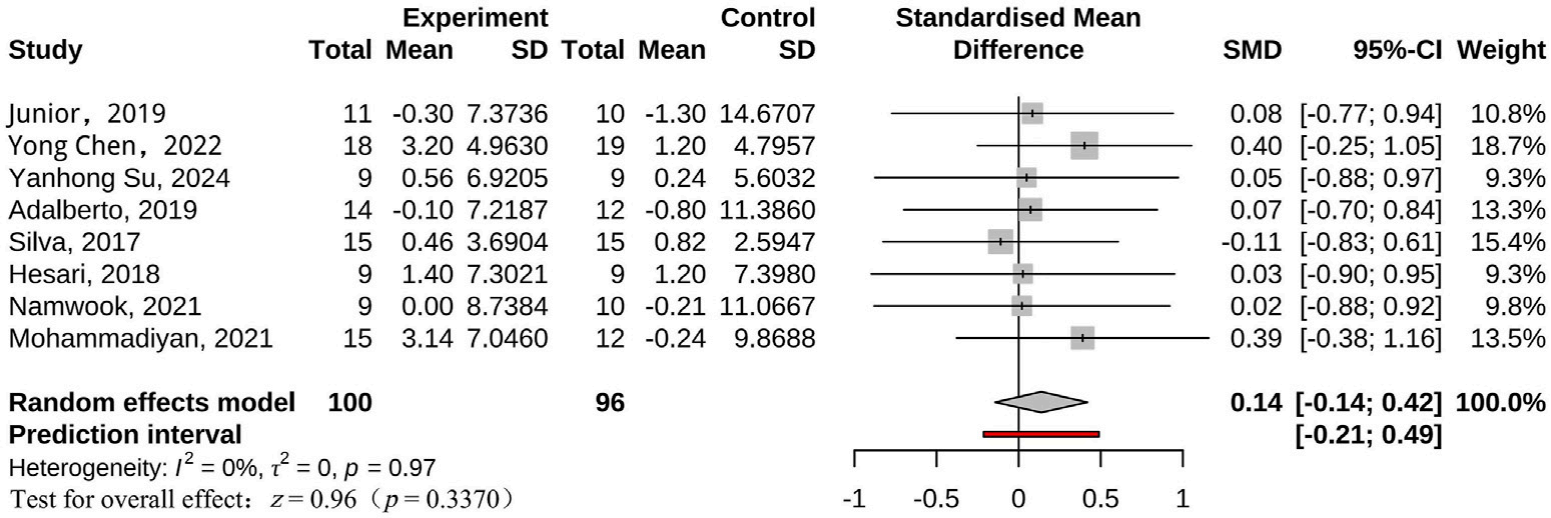
Forest plot for Body Weight.

We found that BFR training significantly reduced BF% (*g* = *0.30* [0.01, 0.58]) in adults with overweight and obesity compared to controls. Further Subgroup analysis revealed no significant difference in improving body fat percentage (*g = 0.20* [-0.20, 0.61] vs. *g = 0.45* [-0.05, 0.95]) between BFR resistance training and BFR aerobic training. In addition, it did not significantly affect body weight (*g = 0.14* [-0.14, 0.42]), BMI (*g = 0.08* [-0.21, 0.38]), waist circumference (*g = 0.13* [-0.28, 0.53]), waist-to-hip ratio (*g = 0.48* [-0.19, 1.15]), fat free mass (*g = 0.06* [-0.31, 0.44]). This contrasts with a previous meta-analysis that reported no improvement in BF% with BFR training in adults with overweight and obesity (Sun, 2022). Although weight loss and the associated reduction in BMI are often primary goals in treating overweight or obesity, our findings indicate that BFR training or control (unrestricted) groups had limited effects on body weight. The mean weight loss ranged from −1.30–3.14 kg in both groups (Figure 8). A meta-analysis of adults with overweight and obesity suggests that achieving clinically meaningful weight loss requires at least 60 min of moderate-to vigorous-intensity aerobic exercise per day (Fogelholm and Kukkonen-Harjula, 2000). Therefore, low-intensity aerobic or resistance exercise alone is insufficient for significant weight loss. Effective weight management relies on maintaining a calorie balance, and combining exercise with dietary control is essential to maximize weight loss and manage obesity effectively (Romieu et al., 2017).

Our study found that both low-intensity resistance training combined with blood flow restriction and low-intensity aerobic training combined with blood flow restriction can effectively improve body fat percentage in adults with overweight and obesity, but did not significantly affect body weight. This could be due to exercise-induced muscle growth offsetting weight loss from fat loss, thereby improving overall body composition. Literature supports this, showing that both aerobic and resistance training effectively reduce body fat while maintaining fat-free body weight without significant weight loss (Willis et al., 2012; Ross et al., 2000; Stiegler and Cunliffe, 2006). In addition, body fat is often more responsive to exercise than body weight, making it a more relevant health indicator for evaluating exercise interventions (Millstein, 2014). For example, a recent meta-analysis reported a greater mean reduction in body fat (MD = −0.39) compared to body weight (MD = −0.30) from aerobic exercise (Kim et al., 2019). Therefore, Aerobic with BFR may be more effective for fat loss than traditional aerobic training, suggesting that low-intensity BFR training can achieve similar fat loss benefits as moderate-to high-intensity aerobic exercise. This highlights its value for managing obesity and improving health in adults with overweight and obesity.

This study demonstrates the potential of BFR training in improving abdominal fat, which is closely associated with cardiovascular health. Abdominal fat is an important indicator of mortality risk and a more effective predictor of obesity-related diseases than body weight or BMI (Moore et al., 2014). Measures of abdominal fat, such as waist circumference and waist-to-hip ratio, are strongly associated with cardiovascular disease risk (de Koning et al., 2007). Research has shown that each 1 cm increase in WC corresponds to a 2% rise in cardiovascular disease risk, while each 0.01 increase in WHR corresponds to a 5% increase in risk (de Koning et al., 2007). Our meta-analysis supports these findings, showing that BFR training significantly reduced waist circumference (*g = 0.13* [-0.28, 0.53]) and waist-to-hip ratio (*g = 0.48* [-0.19, 1.15]) in adults with overweight and obesity. The effects of BFR training on these measures were comparable to, and in some cases larger than, the effect observed without BFR training.

### Practice application

BFR training improves systolic blood pressure and body fat percentage in adults with overweight or obesity. The low-intensity nature of BFR training allows individuals to enhance cardiovascular health and body composition without imposing excessive strain on the body. By activating muscles under lower loads, BFR training is particularly suitable for those with higher body weight, improving training outcomes while minimizing stress on joints and bones. This characteristic also reduces the risk of exercise-related injuries, which is especially important for individuals with limited physical activity who may struggle to adapt to high-intensity training. Several studies in our review suggest that BFR training can be easily incorporated into daily life through low-intensity activities such as walking, cycling, or bodyweight exercises, whether at home or in the workplace. This approach creates more opportunities for physical activity, helping to reduce sedentary behavior and enhancing the practicality and feasibility of BFR training. Combining BFR with “exercise snacks” (Islam et al., 2022; Yin et al., 2024b) at lower intensity performed multiple times daily may further promote cardiometabolic health (Yin et al., 2024c; Yin et al., 2024d) while mitigating the negative effects of sedentary behavior (Yin et al., 2024e; Pinto et al., 2023; Dunstan et al., 2021). However, achieving significant reductions in body weight and BMI likely requires longer durations of BFR training combined with dietary interventions, as exercise alone often has limited effects on weight loss (Swift et al., 2014). Nonetheless, the lack of significant reductions in body weight and BMI does not diminish the potential benefits of incorporating low-intensity BFR training into daily routines.

## Limitations

This study is not without limitations. Firstly, the limited amount of literature available restricted our ability to conduct subgroup analyses. For example, among the studies assessing BF%, six recruited participants younger than 45 years (young adults aged 20–44 and middle-aged individuals aged 45–59) (Kim et al., 2021; Su et al., 2024; da Silva et al., 2020; Chen et al., 2022; Li et al., 2022; Mohammadiyan, 2021), while only one study had participants older than 45 (25). There was also a significant imbalance in the intensity of interventions, with 10 studies using low-intensity BFR training and only one using high-intensity interventions (Li et al., 2022). This uneven distribution hindered more detailed subgroup analyses. Secondly, the lack of standardization in cuff pressure (20–200 mmHg; 50%–80% AOP) and width (5–26 cm) across studies presents another limitation. Future studies should consider that optimal cuff pressure may vary depending on the specific health outcomes being targeted, such as different aspects of cardiometabolic health and body composition. For instance, improving certain indices may require higher pressures. Additionally, cuff pressure should be evaluated alongside exercise intensity, as both factors likely influence the overall efficacy of the intervention (Rossow et al., 2012; Spitz et al., 2019).

## Conclusion

BFR training effectively improves systolic blood pressure and body fat percentage in adults with overweight and obesity, with no significant difference in the effects between aerobic and resistance exercises. Additionally, BFR training did not show significant effects on body weight, BMI, diastolic blood pressure, and V̇O_2_max. This suggests that BFR training can be an effective exercise option for improving cardiovascular health and preventing hypertension in adults with overweight and obesity. Its ability to reduce body fat while maintaining fat-free mass highlights its potential importance in the management of overweight and obesity.

## Data Availability

The raw data supporting the conclusions of this article will be made available by the authors, without undue reservation.
